# Redox properties of ginger extracts: Perspectives of use of *Zingiber officinale* Rosc. as antidiabetic agent

**DOI:** 10.2478/intox-2013-0005

**Published:** 2013-03

**Authors:** Lucia Račková, Máriá Cupáková, Anton Ťažký, Júlia Mičová, Emil Kolek, Daniela Košt'álová

**Affiliations:** 1 Institute of Experimental Pharmacology and Toxicology, Slovak Academy of Sciences, Dúbravská cesta 9, SK-841 04 Bratislava, Slovak Republic; 2 Department of Nutrition and Food Assesment, Institute of Biochemistry, Nutrition and Health Protection, Faculty of Chemical and Food Technology, Slovak University of Technology in Bratislava, Radlinského 9, SK-812 37 Bratislava, Slovak Republic; 3 Toxicological and Antidoping Centre, Comenius University, Faculty of Pharmacy, Odbojárov 10, SK-832 32 Bratislava, Slovak Republic; 4 Institute of Chemistry, Slovak Academy of Sciences, Dúbravská cesta 9, SK-845 38 Bratislava, Slovak Republic; 5 Department of Food Analysis, VÚP Food Research Institute, Priemyselná 4, SK-824 75, Bratislava, Slovak Republic

**Keywords:** *Zingiber officinale* Roscoe, oxidative stress, diabetes, pancreatic β cells

## Abstract

In traditional medicine, several medicinal plants or their extracts have been used to treat diabetes. *Zingiber officinale* Roscoe, known commonly as ginger, is consumed worldwide in cookeries as a spice and flavouring agent. It has been used as the spice and medicine for thousands of years. The present study was undertaken to investigate the potential protective effect of *Zingiber officinale* Rosc. in a model of oxidative damage to pancreatic β cells. The free radical scavenging activities and composition of the isolated n-hexane and ethanolic extracts were confronted with their protective, antioxidant and cytotoxic effects in INS-1E β cells. Unlike the n-hexane extract (exerting, paradoxically, stronger antiradical capacity), both low cytotoxicity and remarkable protective effects on β cell viability, followed by lowering oxidative stress markers were found for the ethanolic extract *Zingiber officinale* Rosc. The present study is the first pilot study to assess the protective potential of *Zingiber officinale* Rosc. in a model of cytotoxic conditions imposed by diabetes in β cells.

## Introduction

Oxidative stress plays a critical role in diabetes type 1 and 2. With regard to their reduced antioxidant reserves, pancreatic insulin producing β cells represent one of its primary targets.

*Zingiber officinale* Roscoe (family, Zingiberaceae), known universally as ginger, is commonly used as a spice and food as well as medicinal agent in Indian, Asian and Arabic traditional medicine in the form of a fresh paste, dried powder, candy (crystallized ginger) or slices preserved in syrup (Ali *et al.*, 2008; Ojewole, 2006). The ginger rhizomes have been used in herbal medicinal practice for the treatment of a range of diseases such as rheumatoid arthritis, hypercholesterolemia, neurological diseases, asthma, stroke, constipation, diabetes or cancer (Lantz *et al.*, 2007).

The research confirmed multiple health benefit of *Z. officinale* rhizome extracts including analgetic and anti-inflammatory effects (Ojewole, 2006; Lantz *et al.*, 2007). Moreover, previous reports point to the therapeutic usefulness of ginger extracts also with regard to diabetes and diabetic complications (Ojewole, 2006; Bhandari *et al.*, 2005). Hyperglycaemia- as well as dyslipidaemia-lowering effects belong to the most known anti-diabetic benefits of ginger.

Hydroethanolic extract of the rhizome ginger is known for its strong free-radical reducing efficacy (Ali *et al.*, 2008). It is mediated mostly by its phenolic constituents that may be divided into two groups: gingerol-, gingeron- and shogaol-related group and diarylheptanoids. A mixture of non-phenolics (sesquiterpene hydrocarbons, carbonyl compounds, monoterpene hydrocabons and esters) contributes to the antioxidative activity and is responsible for the strong aroma of ginger in food, beverages and dietary supplements (Ali *et al.*, 2008).

The essential oil with volatile compounds (prepared by hydrodistillation) showed also a good antioxidant activity with correspondence to its phenolic content (El-Ghorab *et al.*, 2010). Furthermore, antioxidant activity of both volatile and non-volatile fractions has been ascribed to the synergistic effects of phenolics (such as eugenol, shoagols, zingerone, gingerdiols, gingerols, *etc.*). As confirmed by GC-MS, the most abundant terpenes in ginger essential oil are zingiberene, ar-turmerone, curcumene, tumerone and curlone (Singh *et al.*, 2008).

Considering frequent applications of ginger extracts in medicine or food industry, there is a need for better understanding of the commercial drugs composition, based on *Z. officinale* extracts also with regard to their biological efficacies.

In this study, the composition of two different *Z. officinale* extracts (prepared with solvents n-hexane and ethanol) was investigated. Furthermore, their intrinsic antiradical properties with their cytotoxicities and protective effects in a model of oxidative damage to pancreatic INS-1E β cells were compared.

## Methods

### Plant material

The dried powdered roots of ginger were purchased from the local vegetable and fruit market of Vido, Co., Ltd., Bratislava, Slovak Republic (September 2009).

### Chemicals and INS-1E cell culture

DPPH (1,1′-diphenyl-2-picrylhydrazyl) radical was purchased from Sigma Co. (St.Louis, MO, USA) and Folin-Ciocalteu′s phenol reagent from Merck KGaA, Darmstadt, Germany).

INS-1E β cells (kindly provided by Prof. Claes Wollheim, University of Geneva (Merglen *et al.*, 2004) were cultured in RPMI-1640 supplemented with 10% fetal calf serum, 100 U.ml^–1^ penicillin, 100 µg.l^–1^ streptomycin, 2 mmol.l^–1^ L-glutamine, 1 mmol.l^–1^ Na-pyruvate, 55 µmol.l^–1^, 2-mercaptoethanol, 10 mmol.l^–1^ HEPES, pH 7.0–7.4 (KRD molecular technologies, ltd, Slovakia). For assays, the cells were detached by 5–10-min incubation with 0.05% trypsin/EDTA (KRD molecular technologies, ltd., Bratislava, Slovakia).

### Extracts preparation

Air-dried and powdered ginger (*Z. officinale* rhizomes, 2×150 g) was separately extracted with five volumes of n-hexane and 60% aqueous ethanol (v:v), for 48 h, subsequently the solid phase was removed by filtration and combined extracts were concentrated under reduced pressure at 40 °C, to give 10.2 g and 13.2 g crude extract, respectively. The prepared extracts were stored at 4 °C until further analyzed.

### GC-MS analysis

The extracts were submitted to qualitative and quantitative analysis by GC-MS system (Agilent Technologies 7890, Palo Alto, USA) gas chromatograph equipped with an Agilent Technologies 5975C inert XL mass selective spectrometer. The mass range was scanned from m/z 29–420 Daltons.

The non-polar column Agilent 19091B-102 Ultra 2, 25 m × 0.20 mm, film thickness 0.33 µm was programmed from 40–320 °C.min^-1^. Injector temperature was 250 °C, injections 0.5 µl, and split 1:100. The carrier gas (helium) flow was maintained at 0.8 ml.min^–1^ by an electron control of pressure. Identification of the compounds was based on (i) comparison of substance mass spectra with the GC-MS system data bank (NIST 05 library), (ii) comparison of mass spectra with data in the literature (Jain *et al.*, 2007; Jolad *et al.*, 2004).

### DPPH radical-scavenging assay

Free radical-scavenging ability of both extracts tested was determined as described by Wong *et al.* (2006). The capacity to scavenge the lipid soluble DPPH radical was monitored at 517 nm. DPPH methanolic solution (2.9 ml, 1 mmol.l^–1^ solution of DPPH radical solution in methanol) was mixed with the samples tested (0.1 ml) at different concentrations. After 30 min the absorbance was read at 517 nm (Thermo Electron Corporation Genesis 6 Spectrophotometer (UK)). The percentage of absorbance decrease, relative to non-reduced control DPPH solution was evaluated.

### Total phenolic content

The total phenolic content was determined using Folin-Ciocalteu′s method (Lako *et al.*, 2007). Briefly, a 0.5 ml aliquot of extract solution, dissolved in methanol was transferred into the test tube containing 8 ml of distilled water. Afterwards, 0.5 ml of the Folin Ciocalteu′s phenol reagent (1:10 dilution) was added to react completely with the oxidizable compounds or phenolates, and 1 ml of sodium carbonate solution (7.5% solution in water) was added to destroy the residual reagent. The mixture was shaken for 15 s and then left to stand at room temperature for 2 h. The absorbances were measured at 760 nm. Results were expressed as mg.g^–1^ of dry extract. The samples were measured in triplicate.

### Cell viability

Mitochondrial reduction of MTT (3-[4,5-dimethyldiazol-2-yl]-2,5-diphenyl-tetrazolium bromide) was determined spectrophotometrically at λ_max_= 570 nm, following the exposure to H_2_O_2_ or to extracts tested in supplemented medium (1% FBS). Trypan blue uptake *in situ* was determined by exposure of the cells to 0.1% trypan blue in phosphate buffered solution (PBS, pH 7.4, 5 min). The cells were washed with PBS and examined by light microscopy.

### Apoptosis/necrosis detection and caspase 3 assay

The apoptotic changes were assessed by ethidium bromide and acridine orange (EB/AO) staining assay (Ribble *et al.*, 2005; Račková *et al.*, 2009a). Briefly, the cells were grown in 3 cm Petri dishes and treated as described above. EB/AO dye mix (100 µg.ml^–1^ EB and 100 µg.ml^–1^ AO, 20 µl) was added to each dish at the end of incubation time and the cells were viewed under the fluorescence microscope. Each image (50–200× magnification) was collected with XDS-2 fluorescence microscope running standard software. Early and late apoptotic cells were detected by their bright green and orange nucleus with condensed or fragmented chromatin, respectively, whereas necrotic cells were detected by their intact red nuclei, and live cells had a normal green nucleus. For detection of caspase 3 activation, luminiscence assay using Caspase-Glo 3/7 Assay kit (Promega) was performed according to the manufacturer’s protocol. Fifty thousand cells were used for each measurement.

### Intracellular oxidants

Simultaneous oxidation of incorporated 2′,7′-dichlorodihydrofluorescein diacetate (H_2_DCF-DA) and dihydroethidin (DHE) (probes with differing specificity to oxidizing species) was followed in the β cells exposed to H_2_O_2_ stress (Račková *et al.*, 2009a; Halliwell and Whiteman, 2004). In brief, the cells were grown in 96-well plates to a density of 4×10^4^ cells per well and incubated with 15 µmol.l^–1^ H_2_DCF-DA in supplemented phenol red and serum free RPMI-1640 for 30 min at 37 °C. After H_2_DCF-DA loading, the cells were washed and medium was replaced by RPMI-1640 containing 1% FBS and H_2_O_2_ with or without the substances tested. DHE (10 µmol) was added to the wells at the incubation time of 1 hour, followed by additional incubation under the same conditions for 20 minutes. Images were collected by fluorescence microscope and analyzed for reaction intensity.

### Statistical analysis

Each experiment was performed at least three times. Results are expressed as mean value ± standard deviation (SD). Statistical analysis was performed using unpaired Student's t-test using X-Plot v. 2.81 and statistical significance is expressed as *p<0.05, **p<0.01, ***p<0.001 *vs* respective control cells.

## Results

### Determination of total phenolic content

The determined total phenolic content of n-hexane and ethanolic extract amounted to 50.74 and 28.75 mg.g^–1^ of dry extract, respectively. The results are represented as miligrams of gallic acid equivalent (GAE) per one gram of dry extract.

### Radical scavenging activity and composition of extracts isolated from *Z. officinale*

**Figure 1:**
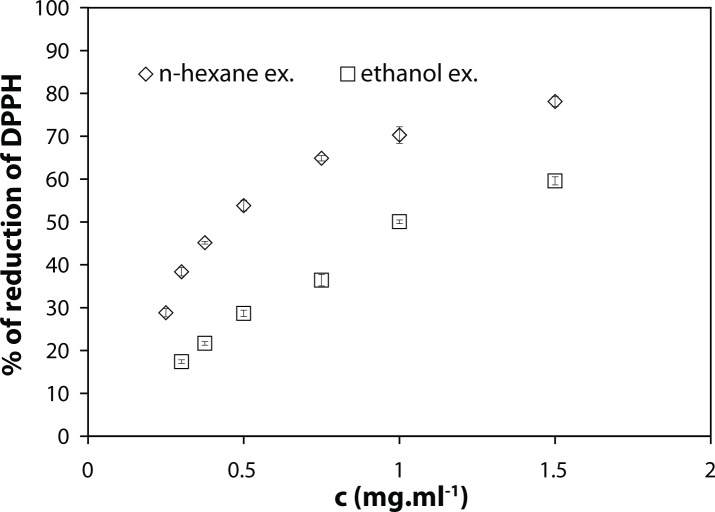
DPPH radical reducing efficacies of the n-hexane and ethanolic extract isolated from *Zinginber officinale* Rosc. Results are expressed as the mean ± S.D., n = 3.

The n-hexane extract showed better reactivity with DPPH ability than ethanolic extract. This was the most evident at the concentration of 750 µg.ml^–1^ of both n-hexane and ethanolic extract tested, causing reduction of DPPH by 64.9±0.6% and 36.4±1.3%, respectively (Figure 1). This outcome was further supported by corresponding IC_50_ values: 1.08±0.04 mg.ml^–1^ and 0.47±0.01 mg.ml^–1^, respectively.

Sample constituents of ethanol extract are given in Table 1: 7-gingerol (16.83%) was identified as the main compound in our analysis, however, 6-gingerol (7.69%) was found as the second major compound. The n-hexane extract profile of ginger (Table 1) shows *alpha*-curcumene as the main compound (19.09%), other major compounds were *alpha*-zingiberene (15.25%), *beta*-sesquiphellandrene (7.163%), *tau*-muurolol (5.00%) and 4-gingerol (4.64%).

**Table 1:** Compounds of *Zingiber officinale* Rosc.

n-Hexane extract		Ethanolic extract	
compound	Peak area [%]	compound	Peak area [%]
borneol	1.25	tumerone	0.49
*tau*-muurolol	5.00	folic acid	0.50
*alpha*-zingiberene	15.25	*ar*-tumerone	1.30
*alpha*-curcumene	19.09	phtallic acic, butyloctyl ester	0.92
*beta*-sesqui-phellandrene	7.16	diepicedrene-1-oxide	2.08
*beta*-cedrene-9-*alpha*-ol	1.37	4-gingerol	0.8
zingerone	1.54	6-gingerol	7.69
4-gingerol	4.64	7-gingerol	16.83
6-gingerol	0.31		
7-gingerol	1.02		
8-gingerol	0.89		

### Cytotoxicity of H_2_O_2_ and extracts tested

**Figure 2:**
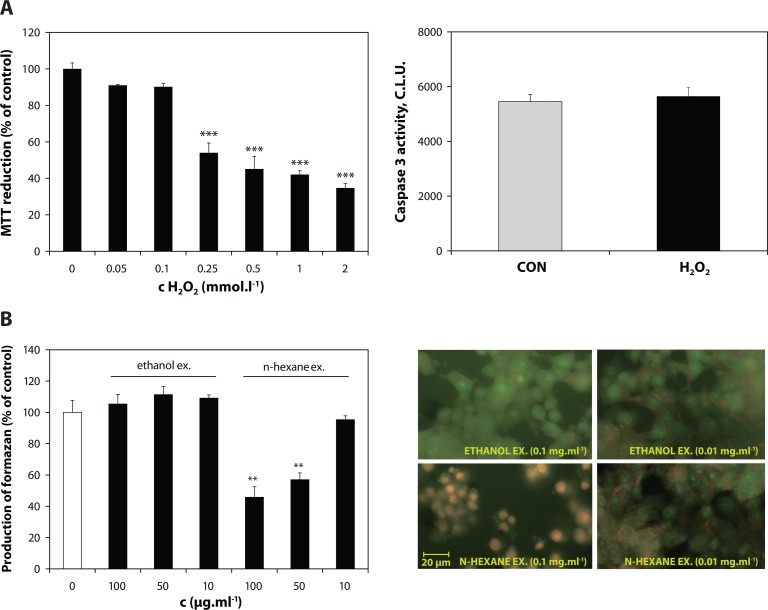
Cytotoxicity of H_2_O_2_ and the *Z. officinale* fractions tested in β cells. **A:** Metabolic activity of INS-1E cells exposed to H_2_O_2_ in medium (1% FBS) for 1 hour. Caspase 3 activity in the cells exposed to 0.5 mmol.l^–1^ H_2_O_2_. **B:** AO/EB uptake and MTT viability of the INS-1E β cells incubated with n-hexane and ethanolic extract for 1 hour. Cytotoxic effect appears as a bright staining of nuclear region of the cells accompanied by membrane blebbing (left bottom image). Results are expressed as the mean ± S.D., n = 3.

Exposure to H_2_O_2_ (0.5 mmol.l^–1^) during 1 hour caused a concentration-dependent decrease of MTT reduction by INS-1E β cells (Figure 2A). Viability decline was further confirmed by positive staining with Trypan blue as well as increased cell permeability for ethidium bromide dye (in AO/EB staining assay). A significant increase in number of the cells with EB-positive intact nuclei was observed following H_2_O_2_ injury. Furthermore, a lack of caspase 3 activation was confirmed within exposure time to H_2_O_2_ (Figure 2A), followed by a drop of caspase 3 activity in the next 2 hours (data not shown). In addition, incubation with H_2_O_2_ (0.5 and 2 mmol.l^–1^) promoted intracellular oxidation preferably of DHE (with a minor portion of H_2_DCF oxidation) (Figure 4).

Unlike the n-hexane extract, causing at the conc. >50 µg.ml^–1^ injury of β cells (Figure 2B), ethanolic ginger extract did not show any notable cytotoxicity at the concentration range tested. The reduction of MTT viability by n-hexane extract was accompanied by nuclear shrinkage and membrane blebbing confirmed by AO/EB staining assay (Figure 2B).

### Reduction of intracellular oxidants and protection of β cells against injury by H_2_O_2_

**Figure 3:**
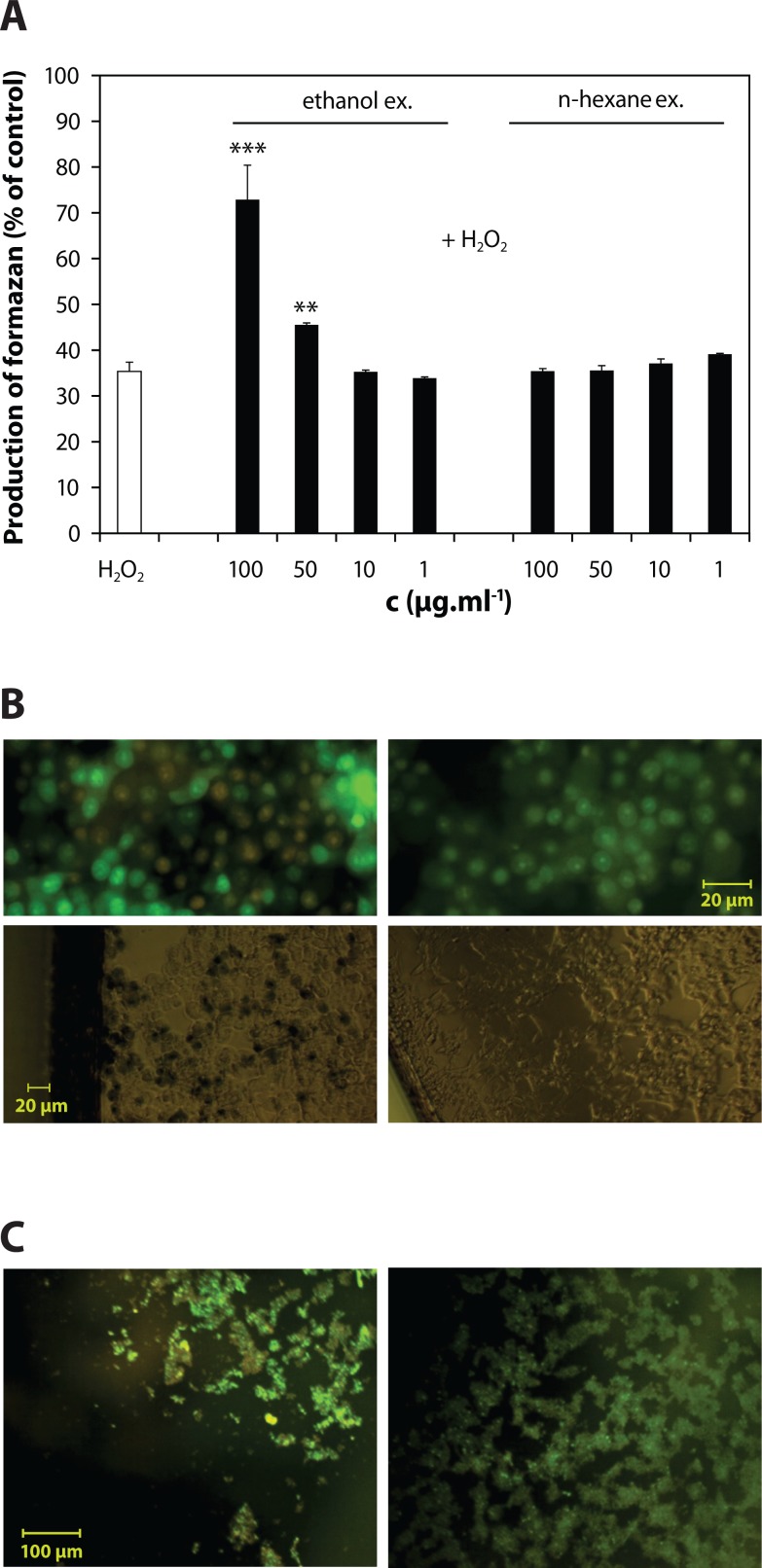
Protection of metabolic activity of INS-1E β cells and *in situ* protective effects of *Z. officinale* ethanol extract. **A:** Significant protection of metabolic activity of H_2_O_2_-stressed cells by ethanolic extract in comparison to lack of efficacy of n-hexane extract. **B:** Protection of viability and **C:** adherence of INS-1E cells by ethanolic extract (0.1 mg.ml^–1^). Dead cells appear as red-fluorescence positive cells. Results are expressed as the mean ± S.D., n = 3.

Only ethanolic extract *Z. officinale* showed a significant dose-dependent protection of metabolic activity of the cells exposed 0.5 mmol.l^–1^ H_2_O_2_ (causing 64.7%-decrease of MTT viability) (Figure 3A). This was accompanied with abolishment of Trypan blue and ethidium bromide uptake by the cells, as shown by microscopic images, suggesting an efficient viability protection (Figure 3B). The loss of cell adherence was also significantly prevented (Figure 3C). The maximum protective effect of ginger ethanolic extract was seen at concentration 100 µg.ml^–1^ (72.9±7.5% of control).

**Figure 4:**
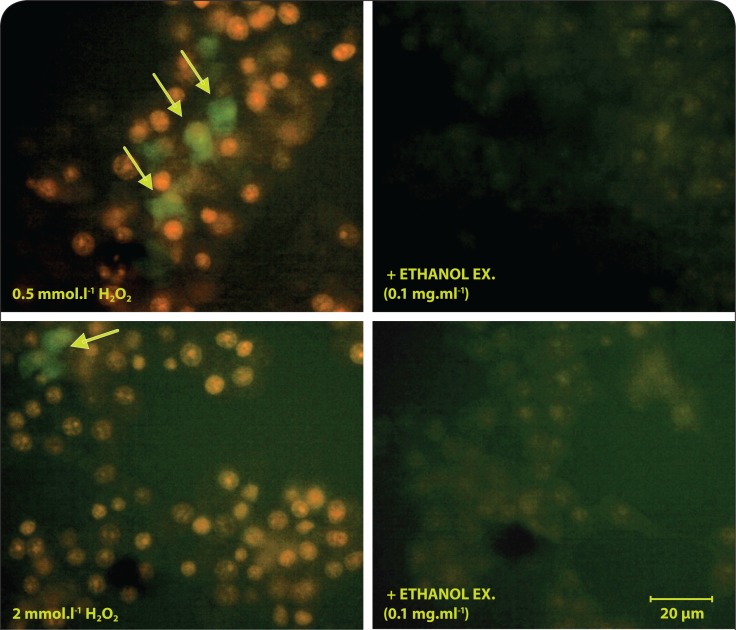
Antioxidant effect of ginger ethanol extract in INS-1E β cells. Fluorescent images indicate the significant prevalence of the cellular DHE oxidation by superoxide, compared to H_2_DCF oxidation by RO•, R = alkyl, alkoxyl, H, NO• (green fluorescence indicated in arrows).

The protective effects of ethanolic extract were accompanied by a significant avoidance of H_2_DCF and DHE oxidation in the stressed cells, appearing as a lack of both ethidium-positive nuclei and dichlorofluorescein fluorescence (Figure 4).

## Discussion

### n-Hexane extract possessed higher content of polyphenols than ethanolic extract

The determined content of polyphenols in ethanolic extract agrees with previously estimated value 21.24 mg GAE.g^–1^ of dry weight of ginger ethanolic extract (Liu *et al.*, 2008). Furthermore, El-Ghorab *et al.* (2010) reported on the value 67.5 mg GAE.g^–1^ of dry n-hexane ginger extract, which is also in good conformity with our result.

### n-Hexane extract showed better radical-reducing capability than ethanolic extract

Phenolic compounds represent a substantial portion of spice antioxidants. Although the oxidative-stress reducing activities of putative antioxidants have been attributed to various mechanisms, DPPH radical-scavenging activity is an appropriate indicator of potential antioxidant efficacy. In analogy with the data reported by El-Ghorab *et al.* (2010), the results of DPPH assay of both extracts tested corresponded to the differences in their phenolic contents. The n-hexane extract showed clearly more potent hydrogen-donating ability than ethanolic extract. However, both determined IC_50_ values are considerably higher than our values reported for substances with strong antioxidant activities – curcumin and *Curcuma longa* extract (3.33 µg.ml^–1^ and 2.34 µg.ml^–1^, respectively (Račková *et al.*, 2009b).

The contents of gingerols as the main phenolic constituents were further confirmed by GC-MS analyses in both extracts tested. Unlike the studies reporting on 6-gingerol as a major compound in the varieties of Zingiber (Ojewole, 2006; El-Ghorab *et al.*, 2010; Jolad *et al.*, 2004), 7-gingerol was identified as the main compound in our analysis, however, 6-gingerol was found as the second major compound. In accordance with reports by Natta *et al.* (2008) and Sacchetti *et al.* (2005), the constituent's profile of n-hexane extract shows *alpha*-curcumene as the main compound, other major compounds being *alpha*-zingiberene, *beta*-sesquiphellandrene, *tau*-muurolol and 4-gingerol. The synergism mediated by a greater variety of phenolics (4-,6-,7-,8-gingerol, 6-paradol, Table 1) in n-hexane extract may account for its better efficacy in DPPH assay (El-Ghorab *et al.*, 2010; Liu *et al.*, 2008). In this regard, the antioxidant synergism of various nucleophiles (represented in the n-hexane extract particularly by secondary and tertiary alcohols, Table 1) with 3,4-dihydroxy-polyphenols has been also proposed (Saito & Kawabata, 2004). This can also explain an increased reactivity of n-hexane extract with Folin-Ciocalteu′s reagent which not only measures total phenols but also reflects the total reducing capacity of a sample.

On the other hand, the actual content of 8- and 10-gingerols (thermally labile compounds loosely detectable by GC-MS) in n-hexane extract could be underestimated (Bilehal *et al.*, 2010). In support of this assumption, both zingerone (retention time (Rt) = 58.27 min) and aldehydes (retention time (Rt) (octanal) = 7.18 min; Rt (decanal) = 14.67 min), decomposition products of thermo-labile higher gingerols were detected in chromatogram of n-hexane extract.

### Hydrogen peroxide caused necrosis in INS-1E β cells

Hydrogen peroxide is a highly reactive compound, present in the majority of the oxidative processes, responsible for pancreatic β cell demise in diabetes type 1 and 2. During insulitis, activated phagocytes can produce as much as 47 nmol of H_2_O_2_ per 10^6^ cells within 30 min corresponding to 1 ml solution of H_2_O_2_ with the concentration of 47µmol/l (Anderson, 1992). In type 2 diabetes, excessive glucose metabolism may lead to generation of superoxide anions, which are spontaneously dismutated to H_2_O_2_ (Nishikawa & Araki, 2007; Leverve *et al.*, 2003). It has been shown that mitochondria are primary targets for H_2_O_2_ damage that may eventually lead to impaired glucose metabolism and decreased insulin secretion (Maechler *et al.*, 1999).

Exposure to H_2_O_2_ caused a decrease of MTT reduction, followed by increase of Trypan blue uptake by INS-1E β cells suggesting both viability decline and injury of metabolic function of the cells (Janjic & Wollheim, 1992). Abundance of EB-positive cells with intact nuclei along with a deficiency of caspase 3 activation suggested that H_2_O_2_ injury caused mainly necrotic death of INS-1E β cells.

### n-Hexane extract exerted increased cytotoxicity in INS-1E β cells

Neither of the ginger substances tested did exert notable cytotoxicities at lower concentrations applied. However, the loss of viability caused by high-dose n-hexane extract was accompanied by presence of apoptotic markers. In paradox, enhanced cytotoxic effects of *Z. officinale* n-hexane extract may be related to its stronger intrinsic redox activities. The cytotoxic effect of natural phenolic compounds has been documented in pancreatic βTC1, HIT as well as INS-1E cell lines, explained by prooxidant effect of these substances (Bortolotti *et al.*, 2009; Lapidot *et al.*, 2002). Furthermore, increased cytotoxicity of n-hexane extract may be associated with higher lipophilicity of its components (Moridani *et al.*, 2003). Correspondingly, zerumbone, a natural cyclic sesquiterpene isolated from *Zingiber zerumbet* Smith (Southeast Asian ginger) has been shown to induce apoptosis in pancreatic carcinoma cells (Zhang *et al.*, 2012). This suggests a promising potential of the n-hexane extract constituents in treatment of pancreatic cancer.

### Ethanolic extract protected INS-1E β cells against injury by H_2_O_2_ along with suppression of intracellular oxidants

Neither H_2_O_2_ nor O_2_^•–^ can oxidize H_2_DCF, but peroxyl, alkoxyl, NO_2_·, carbonate (CO_3_^•–^) and ·OH radicals can, as can peroxynitrite (Halliwell & Whiteman, 2004). Dihydroethidium (dihydroethidine) (DHE) is frequently used as a probe for O_2_^•–^, being oxidized to a fluorescent product, ethidium, which tends to intercalate into nuclear DNA. Apparently, exposure to H_2_O_2_ induced oxidation preferably of DHE (with a minor portion of H_2_DCF oxidation) suggesting that superoxide radicals are the major oxidizing species in the present model. The H_2_DCF can effectively determine the intracellular oxidants only if they are decomposed to radicals, *e.g.* by means of transition metals. The presence of extracellular iron was shown as necessary for the generation of a signal from H_2_DCF upon addition of H_2_O_2_ to the cells (Tampo *et al.*, 2003). Furthermore, the cellular peroxidase level and heme protein content are another determinants for the signal generation from this probe (Ohashi *et al.*, 2002).

Exposure to H_2_O_2_ was shown to result in the depolarization of mitochondrial membrane potential followed by the inhibition of the hyper-polarization effect of glucose (Maechler *et al.*, 1999). In compliance with our result, mitochondrial depolarization was shown to be responsible for the enhancement of superoxide production in many cellular systems (Budd *et al.*, 1997).

Only ethanolic extract *Z. officinale* showed a significant protection of the cells exposed to H_2_O_2_. Its protective efficacy is comparable to our previously reported MTT viability protection efficacy by 68.9% of control, determined for synthetic pyridoindole (a novel antioxidant standard possessing anti-diabetic potential) at its maximum non-toxic concentration (100 µmol.l^–1^) in INS-1E cells exposed to H_2_O_2_ (Račková *et al.*, 2011; Stefek *et al.*, 2002).

The protective effects of ethanolic extract were accompanied by a significant avoidance of both superoxide-dependent DHE- and superoxide-independent H_2_DCF-oxidation in the stressed cells. Accordingly, *Z. officinale* extracts were shown to exert both superoxide and hydrogen peroxide scavenging activity (Khanom *et al.*, 2003; Yang *et al.*, 2009).

In conclusion, the present study suggests that ethanolic extract isolated from *Zingiber officinale* Rosc. is a promising substance for the in depth study of its protective effects against cytotoxic conditions imposed by diabetes in pancreatic β cells. A higher content of phenolic constituents and increased intrinsic antiradical capability of diverse ginger extract preparations may not be obviously related to their improved biological efficacy under conditions of oxidative stress, but, paradoxically, accounts for an enhanced cytotoxicity. In view of INS-1E cells as an insulinoma cell line, however, the remarkable proapoptotic effect of *Z. officinale* constituents may indicate their prospective antitumor efficacies.
